# Idiopathic male infertility – what are we missing?

**DOI:** 10.1080/20905998.2024.2381972

**Published:** 2024-07-22

**Authors:** Luca Boeri, Hussein Kandil, Jonathan Ramsay

**Affiliations:** a Department of Urology, IRCCS Fondazione Ca’ Granda, Ospedale Maggiore Policlinico, Milan, Italy; b Department of Fertility, Fakih IVF Fertility Center, Abu Dhabi, UAE; c Department of Andrology, Hammersmith Hospital, London, UK

**Keywords:** Male infertility, idiopathic infertility, semen analysis

## Abstract

Couple’s infertility is a rising issue worldwide affecting approximately 15% of couples. In 50% of the couples, a male factor infertility can be identified. Moreover, 30% of the men exhibit reduced sperm quality without any identifiable reason, thereby delineating the condition of idiopathic male infertility (IMI). Despite numerous improvements in the diagnosis and treatment of male infertility over the last decades, idiopathic forms are still the most challenging clinical dilemmas. The aim of this article is to describe the comprehensive diagnostic work-up that each idiopathic infertile man should follow. Moreover, potential new pathophysiological mechanisms and suggested treatment options are discussed. A detailed medical history and an extensive physical examination are mandatory to investigate potential treatable causes of MFI. Similarly, standard semen analysis has been proven to be limited in capturing the fecundability of the spermatozoa itself, therefore more advanced examinations, such as sperm DNA fragmentation (SDF) and oxidative stress measurement, are becoming important in clinical practice for IMI. In terms of diagnostic tools, imaging and genetic investigations are useful to classify idiopathic infertile men, however, epigenetic changes have demonstrated to have a role in sperm production and a prognostic value in fertility outcomes. Antioxidant treatment for IMI has been found to be a valid option to counteract ROS action, while gonadotropins are used to improve sperm quality and SDF. Artificial intelligence is promising to better manage idiopathic infertile men in terms of diagnosis and treatment options.

## Introduction

Infertility affects a significant portion of couples during their reproductive years, with nearly 15% experiencing difficulties in conceiving despite regular and unprotected sexual intercourse [1]. Male factor infertility (MFI) accounts for nearly 20% of cases, while a mix of male and female factors contributes to 30% of infertility cases [1]. With up to 50% of infertility cases associated with male factors, clearly emerges how critical is to establish an accurate diagnostic path even in men and implement the most appropriate and tailored treatments [2]. Moreover, emerging evidence have highlighted the need for a more comprehensive assessment of both pure and mixed MFI, enabling personalized management strategies over routine clinical settings [3–5].

Various factors have been attributed to male factor infertility (MFI), but 30% of men exhibit reduced sperm quality without any identifiable reason, thereby delineating the condition of idiopathic male infertility (IMI) [1,6]. The characterization of IMI and its prevalence exhibit consistent variations across the literature [7], contingent upon presumed causal factors and the specific diagnostic protocols employed. It is plausible that, following a more exhaustive diagnostic evaluation, at least one underlying cause of MFI can be identified in four out of five infertile men [8]. Various studies indicates that IMI may be associated with undisclosed morbid diseases that could disrupt the testicular microenvironment and sperm characteristics (e.g. exposure to pollution, reactive oxygen species), resulting in DNA damage and genetic/epigenetic irregularities, thereby diminishing overall sperm quality and fertility potential [6]. Routine semen analysis stands as a pivotal aspect in investigating MFI, significantly correlating with the likelihood of conception [9] [4]. Nevertheless, individual semen parameters are not surrogates for fertility [10]. Indeed, approximately 15–40% of men are infertile despite exhibiting perfect semen analyses, possessing a normal medical history, and undergoing normal physical examinations. Overall, this condition is presently defined as unexplained male infertility (UMI) [11]. This condition differs from IMI, which is characterized by abnormal semen parameters in men without any identifiable reason. A previous study tried to shed light into the clinical differences between IMI and UMI [12]. Corsini et al. analysed data from 3,098 primary infertile men and 107 fertile controls and showed that the prevalence of IMI and UMI was 20% and 5%, respectively [12]. In both of those settings, only minor discrepancies were noted compared to their fertile counterparts. Idiopathic infertile men exhibited lower testicular volume and reduced serum vitamin D levels in contrast to men with unexplained infertility [12]. No additional clinical characteristics proved to be significant throughout the real-world diagnostic evaluations of patients presenting with primary infertility. Thus, it is likely the appropriate moment to move beyond mere disparities in the definition and classification of infertility types and instead invest in technologically advanced (and consequently more beneficial) diagnostic tools to gain a deeper understanding of the pathophysiological mechanisms accountable for the compromised reproductive outcomes [13]. Indeed, at present, current Guidelines still consider basic examinations as the first line diagnostic tools for male infertility [1,4]. However, more advanced tests, such as sperm DNA fragmentation (SDF), epigenetics and artificial intelligence are becoming more popular and important in the everyday clinical practice, particularly in the management of idiopathic male infertility.

Therefore, the aim of this review is to summarize the critical aspect of diagnosis and treatment of idiopathic male infertility, with a particular focus on new discoveries and potential future perspectives.

## The importance of a comprehensive work up–to make a diagnosis

### Personal, medical and physical examination

Medical history should focus on any risk factors that could negatively impact on male’s fertility, being lifestyle, family history (including testicular cancer), comorbidities [14], previous testicular surgery and excluding any potential known gonadotoxin exposure [15]. Congenital or acquired condition affecting testicles’ integrity or function should be evaluated, such as cryptorchidism (uni- or bilateral), history of testicular torsion and/or trauma. Similarly, potential iatrogenic etiologies, including gonadotoxic medications (e.g. anabolic drugs, chemotherapeutic agents, etc.), use of illicit drugs (e.g. marijuana, cocaine, opioids), previous urogenital/pelvic surgery and exposure to radiation, and environmental exposure (e.g. air pollution) should be considered [16,17]. Moreover, compelling evidence has accumulated in regard to the close relationship between overall health status and infertility [18,19]. It emerges that certain medical conditions, such as diabetes, hypertension, obesity, and other non-communicable chronic diseases (NCDs), can significantly impact toward male fertility regardless of age, thus making it imperative to address and manage these health concerns when managing infertility in men. Moreover, infertility per se may increase the risk of developing additional comorbidities compared to the general population [3,20]. Additionally, the observed trend of European males delaying fatherhood over the past two decades, coupled with the rising incidence of health-relevant comorbidities, can further impact men’s fertility [21]. Therefore, systematically collecting a detailed medical history plays a crucial role in patient management. It has been shown that patients with poorer general health exhibit lower sperm concentration, decreased total testosterone (tT) levels, and elevated follicle-stimulating hormone (FSH) values [14]. Moreover, infertile men with abnormal semen parameters are at higher risk of cancer and cardiometabolic disorders [22,23].

A meticulous physical examination is a prerequisite in the assessment of MFI. Secondary sexual characteristics should be evaluated. In cases of congenital testicular deficiency, for instance in Klinefelter syndrome, the clinical presentation is explicit. Due to the intrinsic link between obesity, hypogonadism (primarily functional), and MFI the measurement of body mass index (BMI) and waist circumference is important in all individuals [24,25].

A detailed urological physical examination of the external genitalia represents the mandatory keystone step in a clinical evaluation of MFI. Testes’ size, texture and consistency should be assessed [26]. In routine clinical practice, testicular volume (TV) is measured using Prader’s orchidometer, a reliable and cost-effective proxy for ultrasonography-measured TV [27]. However, uniform reference values for Prader’s orchidometer-derived TV are yet to be established. Previous reports have revealed that infertile men have reduced TV than fertile counterparts, with a linear correlation between TV and tT levels, and a negative one between TV and FSH/luteinizing hormone (LH) [26]. Subsequently, testes consistency should be evaluated, with a focus on palpable abnormalities of the epididymis and of the vas deferens. Potential testicular masses, which may hint at cancer as a linkage between male infertility and testicular cancer is well-established [28]. Specifically, patients with testicular germinal cell tumors (TGCT), prior to cancer treatment, have reported Leydig cell dysfunction, resulting in diminished sperm quality [28].

Reproductive Medicine experts must explore the presence of a clinically significant varicocele, because it has a negative impact on sperm quality [29,30]. Although the diagnosis is primarily made through physical examination, a more detailed investigation with a color Doppler ultrasonography (CDU) can be considered to guide the therapeutic approach. In terms of disease prevalence it has been reported that up to 37.3% of patients seeking initial medical assistance for MFI had clinically diagnosed varicocele [31].

In addition to this, a careful examination regarding the absence of the vas deferens is essential, particularly among azoospermic men [8]. In this context, approximately 26–75% of men affected by unilateral absence of the vas deferens harbor ipsilateral renal anomalies including agenesis [32]. Therefore, abdominal imaging should be offered to men with vas deferens agenesis regardless of the cystic fibrosis transmembrane conductance regulator (CFTR) status to allow for optimal patient counselling.

Physical examination should also include the penis. An accurate assessment of the penis can identify common abnormalities such as phimosis, short frenulum, fibrotic nodules, epispadias, and hypospadias, all of which can contribute to male fertility impairment.

Additionally, a digital rectal examination (DRE) may be useful to rule out prostate abnormalities, this is important in azoospermic men or before initiating any form of testosterone therapy in hypogonadal men who eventually have ended the reproductive path.

### Standard semen analysis: limitations

Semen analysis plays a central role over the diagnostic assessment of MFI, providing important information about macroscopic sperm quality as well as giving useful insights regarding the need for additional testing (e.g. genetic analysis, sperm DNA fragmentation (SDF), etc.) [1]. To ensure consistency and accuracy, the analysis should adhere to the latest WHO criteria reported on the Laboratory Manual for the Examination and Processing of Human Semen (6th edition) [33]. In the 5th edition, data from approximately 1800 men who achieved natural conception within 12 months of attempting to conceive were analysed, with the lower fifth percentile of this data distribution being regarded as the definitive threshold for distinguishing between normal and abnormal sperm parameters. Subsequently, in the 6th edition of the WHO Manual, data from the 5th edition were subjected to further analysis and supplemented with data from an additional approximately 3500 men across 12 countries [33]. It is worth noting that slight disparities in the lower fifth percentile of the data distribution were observed compared to the previous edition. Evidence derived from both the WHO manual itself and clinical practice underscores that the lower fifth percentile of data from men in the reference population does not serve as a definitive demarcation between fertility and infertility [10]. Indeed, infertility is a complex diagnosis that takes into account several factors thus including the partner and several men’s characteristics therefore semen analysis should be interpreted as decision limits rather than reference values. Nevertheless, the classification of sperm parameters as normal or abnormal (based on the 5th percentile) remains of utmost clinical importance in everyday management protocols. Present guidelines endorse the use of sperm quality severity to determine the necessity for diagnostic assessments and to propose potential treatment strategies for male factor infertility (MFI). Indeed, a recent study revealed that the adoption of updated reference criteria for semen parameters by WHO-2021 has resulted in a reclassification of the severity of semen abnormalities compared to previous editions [34]. More in details, one in three infertile men demonstrated a deterioration in semen categorization according to WHO-2021 vs. WHO-2010. These men displayed more severe clinical, hormonal, and SDF index characteristics. Additionally, ART outcomes were lower for men with worsening sperm abnormalities per WHO [35]. Consequently, WHO [35] criteria appear to identify a subset of patients (with abnormal semen parameters) more accurately, who were previously considered ‘normal’ according to WHO-10 parameters [29].

Despite having a fundamental role in the baseline diagnostic work-up of each infertile man, standard semen analysis only represents a macroscopic evaluation of sperm quality. Since 41% of fertile men and 12% of infertile men exhibit normal macroscopic/conventional sperm parameters, conventional semen analysis per se may not provide sufficient accuracy in the setting of male infertility diagnostic work-up [10]. Indeed, the diagnostic efficacy of conventional semen analysis is constrained by the absence of information regarding the functional status of spermatozoa, which is closely linked to their actual fertilization potential. For these reasons, advanced sperm tests are becoming of critical importance in the management of male infertility.

### Sperm DNA fragmentation: role and test

Sperm DNA fragmentation is a molecular marker of sperm chromatin health. As previously mentioned, conventional semen analysis is limited in its ability to capture the functional and molecular aspects of spermatozoa, such as fertilization potential and DNA or chromosomal integrity. Therefore, SDF and its diagnostic utility for male infertility have captured the interest of many reproductive scientists and clinicians worldwide.

Several risk factors for MFI (higher levels of systemic inflammation and signs of metabolic diseases) have been shown to be correlated with higher SDF levels [36,37]. Elevated levels of sperm DNA fragmentation (SDF) have been identified in men with unexplained or idiopathic infertility, as well as in conditions commonly associated with infertility, such as varicocele [36]. Furthermore, increased SDF values are linked to recurrent pregnancy loss (RPL), advanced age, the frequency of miscarriages, and outcomes of assisted reproductive technology (ART) pregnancies [38,39].

Conversely, other Authors failed to find an association between elevated SDF valued and RPL [40,41].

Recognizing the demand for advanced sperm testing, SDF assessment was recently incorporated into the sixth edition of the WHO laboratory manual for the examination and processing of human semen, within the ‘Extended Semen Examination’ section (‘World Health Organization (WHO) WHO laboratory manual for the examination and processing of human semen. 6th ed. Geneva [35]: ’ n.d.). However, the manual does not offer specific guidance on the methodology to be employed, and the absence of a definitive cutoff in the literature necessitates the use of laboratory-specific reference limits. In addition, despite increasing evidence regarding the utility of SDF in clinical practice, there are limitations and inconsistency across international Guidelines in identifying primary infertile men that would deserve this investigation [42].

Several methods for detecting SDF are available, thus including the TUNEL assay [43], the comet [42], the SCD [42] and the SCSA [44].

According to a recent survey among male infertility experts, the TUNEL appears the most widely used worldwide [42]. However, results might be influenced by the availability of instrumentation and dedicated personnel.

One important issue of SDF is the lack of a standardized cutoff. Santi et al. proposed a 20% threshold to differentiate infertile vs fertile men [45]. This threshold has also been endorsed by Agarwal et al. in their clinical guidelines [36].

Overall, expert recommendations suggests that SDF testing method should consider factors such as resource availability, personnel expertise, and laboratory complexity. While there is a lack of standardized cut-off values, each laboratory may establish its reference values based on predictive values for fertility outcomes. Nevertheless, a 20% cut-off is commonly utilized in clinical practice [42]. Clear indications for SDF testing vary among current guidelines but generally include unexplained male infertility (UMI) and idiopathic male infertility (IMI), recurrent pregnancy loss (RPL), clinical varicocele, the presence of known risk factors, recurrent assisted reproductive technology (ART) failure, and in some instances, ART planning [46].

### Oxidative stress: role and test

Oxidative stress has emerged as a significant mechanism contributing to IMI. Previous research indicates that spermatozoa with morphological abnormalities are susceptible to generating excessive reactive oxygen species (ROS) while exhibiting reduced antioxidant capacity [6]. Moreover, oxidative stress is frequently observed in idiopathic infertile men, characterized by an imbalance between ROS levels and antioxidant capacity compared to fertile males [47]. Nonetheless, this association has not been found in a recent study by Rasmussen et al., where OS was not different between infertile and fertile men [41]. Elevated OS can negatively impact fertility through various pathways. OS can induce the formation of mutagenic or genotoxic by-products in germ cells and spermatozoa that may result in a negative impact on spermatogenesis, semen parameters, semen quality, fertilization, pregnancy, and health consequences for future progeny [48].

Despite the growing recognition of oxidative stress’s role in male infertility over recent decades, consensus has yet to be reached regarding which patients should undergo screening for OS or which tests should be conducted to assess ROS levels in semen samples. Additionally, controversy persists regarding the type, dosage, and duration of antioxidant therapy for patients exhibiting excessive ROS levels [49].

Direct and indirect methods are available for ROS measurement. Myeloperoxidase, 8-hydroxy-2-deoxyguanosine, thiobarbituric acid reactive substances test and total antioxidant capacity (TAC) are indirect methods that evaluate the extent of ROS-induced adverse effects. Conversely, chemiluminescence, dihydroethidium probe, nitroblue tetrazolium test (NBT), electron spin resonance, and cytochrome c reduction analysis can directly detect ROS in seminal fluid [49].

Additionally, oxidation-reduction potential (ORP) captures oxidative stress by reflecting the balance between oxidants and antioxidants, and demonstrating a strong correlation with semen quality [6]. The Male Infertility Oxidative System (MiOXSYS) represents a novel and user-friendly system utilized for assessing ORP in human semen [50]. Studies have revealed that semen from male partners of fertile couples typically exhibits lower ORP levels compared to those from infertile male partners, with higher SDF observed in infertile men versus controls [6].

Given the limitations of conventional semen analysis, ORP is proposed as an additional clinical biomarker for Male Oxidative Stress Infertility (MOSI) in men with abnormal semen analysis and male infertility. Infertile men with MOSI are recommended to undergo comprehensive evaluation to identify treatable causes and initiate appropriate therapy, including antioxidants or hormonal interventions, to mitigate oxidative stress [6]. Conversely, infertile men without MOSI are advised against antioxidant therapy. Thus, the measurement of ORP and the stratification of male fertility/infertility based on ORP represent crucial tools in managing infertile couples. However, current guidelines do not advocate systemic testing for ORP in infertile men [1], thereby reducing their integration into clinical practice. Indeed, a recent survey among reproductive specialists reported a low utilization of OS testing in routine clinical practice [48]. This was mainly due to lack of consensus on appropriate tests and their clinically relevant cut-off values, and an absence of standardization of laboratory techniques. Although sensitivity and specificity of various tests have been published, they remain variable, non-standardized and without general diagnostic recommendations. Moreover, numerous testing methods have been developed over the past few decades to determine OS or measure ROS in semen [51]. However, these tests currently have limited practical use and are mostly limited to research [48]. Given these uncertainties, clinical practice guidelines on OS testing and the use of antioxidant therapy in the management of the infertile male are urgently needed.

### The role of MAGI in male infertility

Male accessory gland infections (MAGI), refers to inflammatory or infectious diseases of the prostate, seminal vesicles and Cowper’s glands. It is important to distinguish MAGI from the broader category of male genital tract infection/inflammation (MGTI), which encompasses the overall genital tract. Typically, a high presence of leukocytes and/or pathogens in semen, along with signs of inflammation in the male genital tract, indicate the presence of MGTI. MAGI ranks as the third most common cause of male infertility, following idiopathic infertility (28.4%) and varicocele (18.1%), although previous reports have indicated a prevalence of up to 36.7% [52]. Infections caused by Chlamydia trachomatis, Escherichia coli, and Neisseria gonorrhoeae are among the most common culprits, leading to an excessive accumulation of leukocytes within the male genital tract.

Regarding diagnosis, after ruling out urinary tract infections (including urethritis), an inflammatory process is indicated by the presence of over 10^6^ peroxidase-positive white blood cells (WBCs) per milliliter of ejaculate. In such cases, semen culture or PCR analysis should be conducted to identify common urinary tract pathogens. A concentration of over 10^3^ colony-forming units (CFU) per milliliter of urinary tract pathogens in the ejaculate suggests significant bacteriospermia [53].

However, the existing guidelines lack clarity regarding the timing, settings for semen culture, and specific germs to target. Specifically, according to strict dictates of science deriving from the most rigorous literature, the EAU guidelines suggest performing a semen culture when leukocytospermia is present, which is indicated by >10^6^ peroxidase-positive white blood cells per milliliter of ejaculate, possibly indicating an active ‘infection-driven’ inflammation [1]. Likewise, the AUA/ASRM guidelines, suggest that routine semen cultures have not been prospectively demonstrated to benefit infertile couples, therefore, screening for infection is not needed unless pyospermia is present [4]. To this aim a cross-sectional study involving 523 white-European infertile men revealed that high leukocyte levels in semen did not always indicate an underlying bacterial infection [54]. Consequently, when validating the EAU guidelines, it led to an 80% failure rate in detecting infected semen cultures and conducting 120 unnecessary examinations.

Moreover, it is also not clear which pathogen should be tested in semen samples. Asymptomatic semen infection is a common occurrence among men seeking medical assistance for primary infertility, with one in five men being affected irrespective of leukocyte counts [55]. The frequently isolated pathogens include Ureaplasma Urealyticum, Enterobacteriaceae spp, human papilloma virus (HPV) (any), Mycoplasma hominis, and Chlamydia trachomatis, many of which are not typically detected through standard semen cultures. Several studies has indicated that the presence of a positive semen culture is intrinsically linked to impaired sperm concentration and reduced progressive sperm motility, especially in cases involving Ureaplasma, Mycoplasma, and HPV [55,56], therefore their testing in the diagnostic workup of infertile couples is important.

Several pathophysiological mechanisms have been proposed to explain the impact of MAGI on male infertility. First, MAGI might be responsible for production of ROS and/or inflammatory cytokines. Consequent oxidative imbalance might lead to peroxidative damage of spermatozoa, decrease motility, acrosyn activity, hyperviscosity and altered SDF. Second, impaired secretory capacity of the accessory glands and reduced production of substances that promote sperm maturation has been linked with MAGI. Third, MAGI may lead to anatomical obstruction or sub-obstruction of the seminal tract and finally, a direct effect of pathogens on sperm quality has been proposed [53].

Despite various reports, a definitive management strategy for MAGI remains elusive. Antibiotic treatment was proposed for leukocytospermia associated with male infertility. Additionally, antioxidants capable of reducing reactive oxygen species (ROS) generated by semen leukocytes have been utilized in patients with leukocytospermia. However, consensus regarding the efficacy of each treatment or the necessity of treating leukocytospermia is lacking [52]. Although not univocal results exist, a recent review showed that antibiotics may ameliorate semen quality and pregnancy rates. However, the quality of evidence is insufficient to draw definitive conclusions [57].

### ASA: role and test

Antisperm antibodies (ASA) are immunoglobulins targeting antigens present on the sperm surface. When the blood-testis barrier is compromised due to injury or illness, mature germ cells become exposed to the immune system, leading to the development of ASA [58]. ASA may impact sperm motility, acrosome reaction, capacitation and fertilizing ability. However, the indication and the clinical impact of ASA are not clear. Moreover, the prevalence of ASA in infertile vs fertile men is still a matter of debate [59]. For this reason current guidelines do not suggest ASA testing at baseline diagnostic work up of infertile men [1,4].

ASA can be tested by direct or indirect methods. The mixed antiglobulin reaction (MAR) test is a direct method performed on fresh samples and the immunobead (IB) test on washed spermatozoa [58]. The indirect test is used to measure sperm-specific immunoglobulins in sperm-free fluids such as seminal plasma, heat-inactivated serum and dissolved cervical mucus.

Results from a recent survey among infertility experts showed that indications for ASA test included sperm agglutination, asthenozoospermia or failed ART cycles [58]. The majority of participants recommended low dose steroids as the first-line therapy for patients who had positive ASA testing, whereases 30% suggested ART [58].

### Peripheral lab tests: hormonal evaluation, lipid profile, and others

Male hypogonadism is frequently found in infertile men, with several distinct phenotypes, each reflecting an underlying disease characteristic [1]. Infertile men may present with either secondary or primary hypogonadism [60]. According to the circulating levels of tT, FSH and LH men can be classified into different types of hypogonadism. Notably, primary hypogonadal men face a significantly heightened risk of azoospermia (24-fold increase) and small TV (13-fold increase) compared to eugonadal men, thus portraying the most unfavorable clinical scenario in terms of impaired fertility [61]. Moreover, it has been observed that median levels of sex-hormone-binding globulin (SHBG), which influence the availability of testosterone, tend to rise across age quartiles while decreasing in concurrence with increases in BMI [62].

Therefore, the inclusion of a comprehensive evaluation of sex hormones levels is imperative over the diagnostic work-up of MFI, thus including tT, FSH and LH. Given the significant impact of age and BMI on SHBG levels and the subsequent availability of testosterone, it is crucial to consider these factors when formulating a management strategy for infertile men with hypogonadal conditions.

Additionally, given the higher rates of cardiometabolic conditions of infertile vs fertile men [18], the inclusion of lipid profile (glucose, cholesterol, triglycerides) should also be considered.

### Imaging in infertile men

Scrotal color Doppler ultrasound (US) provides valuable information for the clinical management of varicocele. Physical examination is the primary method for varicocele evaluation, but scrotal Doppler US allows for more accurate and reliable assessment of venous reflux and diameter, helping the decision-making process for treatment [63]. Moreover, scrotal Doppler US plays a crucial role in the evaluation of testicular masses, particularly in infertile men with an increased risk of testicular cancer. Studies have shown that infertility may be associated with an elevated risk of testicular malignancies, making the accurate assessment of testicular masses of paramount importance [18]. Moreover, for infertile men at increased risk of testicular cancer, scrotal US offers a non-invasive and highly sensitive method for early detection and differentiation of benign and potentially malignant masses, potentially leading to improved treatment outcomes and fertility preservation.

Obstruction is suspected in men with low seminal volume, acidic pH and severe oligozoospermia or azoospermia. In these cases, scrotal US and transrectal ultrasounds have proven clinically valuable for detecting absence of the vas deferens [64].

### Genetic tests

Current EAU guidelines recommend that infertile men undergo a karyotype analysis (KA) when azoospermia or oligozoospermia (spermatozoa <5 million/mL) is detected [1]. KA is also indicated if family history suggests repeated spontaneous abortions, malformations, or intellectual disability. In addition, the AUA/ASRM guidelines, suggest performing KA and Y-chromosome microdeletion analysis in azoospermic or severe oligozoospermic men (<5 million sperm/mL) with elevated FSH or testicular atrophy or a presumed diagnosis of impaired sperm production as the cause of azoospermia [4]. However, KA is not always sensitive enough when applied to such sub-category of MFI men. Therefore a nomogram based on LH values, mean TV, and sperm concentration was proposed to improve the detection of chromosomal abnormalities [65].

Moreover, current EAU guidelines suggest testing men for Y-chromosome microdeletion if sperm counts are <5 million sperm/mL and recommend a mandatory analysis only if <1 million sperm/mL are found [1]. Likewise, the AUA/ASMR guidelines suggest testing for Y microdeletion in azoospermic men or severe with severe oligozoospermia (<5 million sperm/mL). On the other hand, CFTR testing is suggested by EAU guidelines, in any patient with unilateral or bilateral absence of the vas deferens or with documented seminal vesicle agenesis. Instead, the AUA/ASRM suggest CFTR testing in men with vasal agenesis or with any idiopathic obstructive azoospermia [4].

## Summary of recommendations for the diagnostic work-up for idiopathic infertile males

As previously stated, the identification of an underlying cause of MFI is strongly related to the quality and accuracy of the diagnostic work-up. For this reason, current Guidelines recommend that each infertile man is evaluated with a detailed medical and sexual history as well as a meticulous physical examination with particular attention on the external genitalia. At least two consecutive semen analyses, performed according to the indications of the latest WHO Laboratory Manual for the Examination and Processing of Human Semen (6th edition), should be requested. To investigate the hormonal regulation of spermatogenesis, a comprehensive evaluation of sex hormones is imperative over the diagnostic work-up of MFI, thus including tT, FSH and LH. Scrotal US might be useful in idiopathic infertile men to role out testicular masses and for a more precise grading of varicocele severity. Since MAGI have been frequently found associated with impaired sperm quality, a semen culture should be performed in men with leukocytospermia. In idiopathic MFI, more advanced tests are useful to guide treatment options, thus including SDF and oxidative stress. Elevated SDF have been found in IMI and several treatment options can be suggested in this scenario, among which antioxidants and hormonal treatment are the most widely used. Similarly, the measurement of oxidative stress can be useful for the stratification of male fertility/infertility based on ORP, once again suggesting potential treatment options to reduce ROS levels. Finally, genetic tests should be performed in IMI according to the current guidelines.

## Increasing understanding of idiopathic male infertility

### Genome instability

Over the past century, male fertility has been experiencing a steep decline, with a study in 1992 which was done on 61 articles including 14,947 males demonstrating this significant finding over the last 50 years [66]. Attempts made to explain this decline have been inconclusive, yet an interplay between internal factors namely, genetic, epigenetic and environmental and lifestyle factors have been considered [67]. While attempting to decipher the underlying pathology behind the IMI-related seminal abnormalities, genetic alterations are highly suggested, possibly justifying the increased morbidity associated with male infertility, hence spermatogenic abnormalities could be identified as a local manifestation of a more systemic disorder [67]. Our current understanding entails the presence of genetic background playing a pivotal role in the pathogenesis of the many forms of male infertility, yet many relevant genetic entities remain unavailable in clinical setting, rendering nearly 40% of male infertility falling under the idiopathic category [68]. Despite this fact, male infertility based genetic studies have highlighted on more than 500 relevant target genes, but still lack a solid understanding regarding the function of their relevant proteins in the causation of infertility [69]. Sperm maturation is a vital step during spermatogenesis, requiring a crucial process wherein histones are replaced with protamine molecules. This would subsequently render chromatin more tightly coiled and protected, ultimately contributing to sperm maturity. Furthermore, genetic instability may arise amid DNA replication and repair, resulting in gross chromosomal rearrangements, copy number variants (CNVs), single nucleotide variants and aneuploidy, which contribute to genetic modification [67]. Interestingly, a study by Punjabi et al., which investigated the degree of genetic instability comparing between fertile patients and their counterparts of sub fertile normozoospermic and non-normozoospermic patients, by assessing DNA fragmentation and chromatin integrity, found that sub fertile normozoospermic patients had significantly lower degree of chromatin decondensation compared to fertile group [70]. Kothandaraman et al., proposed that sperm and seminal fluid of infertile individuals demonstrate variable patterns in the relevant genetic structures including ROS genes, antioxidant genes and single-nucleotide polymorphisms, hence suggesting that relevant genetic and proteomic factors govern the pathogenesis of the reactive oxygen species (ROS) induced IMI [69]. Furthermore, genetic instability could result from aneuploidy that is defined as chromosomal numerical abnormalities, which is not only considered a common cause of genetic instability, but also an outcome [71]. Moreover, in the general population, reciprocal translocation occurs in 0.9/1000 newborns, while Robertsonian translocation occurs in 1/1000 newborn, both of which are seven- and nine-times higher in incidence in infertile males, respectively. Aside from aneuploidy, copy number variants which is a molecular abnormality consisting of genomic repetition that could be linked to male infertility as in the AZF microdeletion of the Y chromosome. Sperm DNA replication and cellular division is a process that could display a wide range of random genetic mutations, knowing that the mutational single base substitution rate is approximately ~ 1–1.5×10–8 per generation. Advanced paternal age at conception is believed to contribute to increased incidence of offspring’s germline de novo mutations [72], and to further complicate the matter, sperm DNA repair occurs exclusively during the early stages of spermatogenesis, and relies afterwards on the oocyte to perform the DNA repair. Loss of sperm DNA integrity is detrimental to sperm function, and the most severe form is the double-strand DNA breaks (DSBs), which may take place as a result of compromised function of topoisomerase II taking place during the different transcription stages. Alternatively, failure to repair the endogenously occurring DSBs during the meiotic recombination, may result in genetic instability, which could lead to infertility secondary to apoptosis and genetic anomalies [73].

### Telomere length and male fertility

In an attempt to identify molecular markers to assess male fertility, an association between sperm telomere length and spermatogenesis has been observed. Shorter sperm telomeres were associated with altered semen parameters [74]. Telomeres are non-coding structures that are located at the eukaryotic chromosomal ends serving as protection against entanglement of genetic structure. In such context, telomeres tend to decrease in length with ageing resulting from cell division, genetic susceptibility or exposures to genotoxic hazards. They are considered crucial in maintaining of the genomic integrity during cell division, this occurs through masking the chromosomal ends so as not to prevent end-to-end fusions, or to be identified as double-strand DNA breaks that could result in initiation of damage response [75]. Telomeres are nucleotide repeats that cap the chromosomal ends, offering genomic integrity and tend to shorten with every cell division. They are common site for oxidative damage due to their high concentration of guanine, yet with the help of telomerase enzyme, with its two subunits namely, telomerase reverse transcriptase and telomerase RNA component, telomere shortening, and loss of DNA is minimized along the process of cell division [76]. Being repressed in most somatic cells, telomerase activity promotes the stem, embryonic and germ cellular proliferation. Furthermore, telomerase activity appears to decline along the course of sperm maturation, whereby spermatogonia appear to exhibit the highest activity, while spermatocytes and spermatids expressing much lower activity and its complete absence in the epididymal sperm. Several studies have demonstrated an association between a short sperm telomere length and idiopathic male infertility, demonstrating a positive correlation between sperm parameters and telomere length, and a negative correlation with sperm DNA fragmentation [77], yet other studies on the other hand have failed to demonstrate any association between sperm telomere length and sperm parameters [78]. In a systematic review and meta-analysis by Yaun et al., 12 observational prospective cohort studies have been investigated with about 1700 patients being enrolled and have demonstrated a positive correlation between the sperm telomere length and sperm parameters and have found that cut-off length is 1 which had a sensitivity and specificity of 80% [79].

### The significance of the seminal plasma microbiome

Recent innovative measures assessing male fertility have witnessed notable breakthrough especially after the adoption of Next Generation Sequencing, which offered a more comprehensive and detailed analysis of the microbial population in an entire sample [80]. Since the number of microorganisms comprising human microbiota surpasses the human cells at a 10 to 1 ratio, the human microbiome has become a field of interest in an attempt to unravel potential clues deciphering idiopathic male infertility. One of the reasons behind this attentive focus on human microbiome lies in the fact that a hypothesis claims a connection between human gut microbiome and reproductive health, and is entitled gut-testicular axis, moreover, an association has been demonstrated between altered human gut microbiome and changes in reproductive hormones and spermatogenesis [81,82]. This could possibly be explained by the systemic inflammatory reaction induced by altered gut microbiome, resulting in an increased level of oxidative stress, hence explaining the role of probiotic antioxidant therapy in the management of male infertility [81]. Studies have demonstrated the impact of the different microbial species on semen parameters, so for instance, unlike the negative impact of Ureaplasma urealyticum and Mycoplasma hominis on the semen parameters, Lactobacillus appear to offer a positive and protective impact [83,84]. In a study by Garcia-Segura et al., a negative correlation was observed between bacterial strains and sperm DNA fragmentation [85]. Several studies have shown a positive effect of probiotics on sperm quality, thus including sperm concentration, motility and normal morphology [81]. This may be associated with the direct effect of probiotics on spermatogenesis and maturation process or indirectly by removing the adverse effects of obesity, promoting hormonal balance and increasing the level of total antioxidant capacity [86].

### Enhancing Diagnosis through Y Chromosome Interrogation: Avenues with Artificial Intelligence

One of the common genetic causes behind spermatogenic failure is Y-chromosome microdeletion (YCMD) especially the AZFc deletion, which occurs in 80% of cases followed by less frequent forms including AZFb and AZFa [87]. Yet, when it comes to the impact on sperm production, complete deletion of the AZFa located on the proximal region of Y chromosome (Yq11), appears the most severe, being associated with sertoli cell-only syndrome. In a study by Kim et al., nearly 11% of 1,226 infertile patients had evidence of YCMD, which was also detected in patients with NOA and severe oligozoospermia in 14% and 20% of the cases, respectively [88]. In another study, 81 infertile patients presenting with variable idiopathic seminal abnormalities including azoospermia, oligoastheno- and oligospermia, YCMD was detected in 6.17% of the patients [89]. It’s worth mentioning that sperm with de-novo deletions may occur following environmental and genetic factors, resulting in pregnancy with child with deleted Y chromosome, emphasizing on the importance of investigating the male’s family history and environmental exposures [90]. Screening for YCMD, whenever indicated is of significant value, especially in the context of ART, knowing that Y chromosomal abnormalities are inheritable to the male offsprings, hence perpetuating the infertility to subsequent generation.

### Unraveling the Male Factor in Euploid Embryo Failure

The transcriptional activity of the spermatozoa, which is the paternal contribution to the developing embryo, remain inert despite being highly differentiated, and functions as a vehicle for the paternal genetic material in the process of embryonic development. The unique genetic structure of this haploid cell is achieved through the process of histone to protamine replacement that results in potent DNA packaging, achieving a significant level of compaction, reaching 10% the somatic cell nuclear volume. Moreover, this process results in nuclear modification that facilitates sperm transit, while offering more protection to the sperm in the female genital tract [91]. For this compacted genetic structure to maintain its stability, the protamine unit that replaces histone is smaller and rich in cysteine, which offers the desired genetic stability through its disulfide cross linkage. If the protamination process fails, the protective effect offered by the aforementioned structural changes will not take place, rendering sperm DNA more prone for damage, resulting in either single- or double-strand DNA breaks, which if not repaired by the oocyte regenerative process, abnormal embryonic development may take place. It was observed that that elevated SDF has been associated with the presence low levels of sperm protamine [92]. Furthermore, ART-related outcomes from fertilization to pregnancy and embryo development are positively correlated with sperm chromatin development and integrity [93]. Yet, other studies failed to demonstrate the association between chromatin maturity and embryo development. Furthermore, infertile males appear to harbor abnormal levels of protamine 2 (P2) as demonstrated in a study by Carrell et al., which demonstrated in a cohort of 75 patients presenting for IVF treatment, 17% had no measurable P2 compared to fertile controls (*p* < 0.005), with further evidence of decreased sperm penetration, normal morphology and progressive motility (*p* < 0.005), yet, 50% of patients with no measurable P2 had successful ICSI-pregnancy [94]. It was shown that, unlike fertile males, where P1 and P2 are nearly equal, the P1: P2 ratio in infertile males appears abnormal.

## Treatment of idiopathic infertility

### Transforming Lifestyle for Male Fertility: Reality or Myth?

With an increasing interest in investigating the different lifestyle factors impacting male reproductive health, it is crucial to explore a possible relationship between stress and male infertility, which includes but is not limited to the physical and emotional factors [95]. Modern lifestyle is highly linked to stressful exposures that are associated with male subfertility. Few studies have demonstrated the link between stress and male fertility, underlining a potential relationship between both entities with evidence of low levels luteinizing hormone (LH) and testosterone and subsequent decline in sperm count and quality among patient exposed to high levels of stress. Other lifestyle factors involve dietary patterns, which are highly linked to male reproductive capacity, testicular function and sperm quality [96]. Obese males for instance, tend to harbor higher incidence of oxidative stress, which is a common cause for abnormal semen parameters and sperm DNA fragmentation [97]. Other exposures including use of tobacco and alcohol intake have been also linked to suppressed spermatogenesis [98]. Increased physical activity is associated with better sperm parameters and reduced oxidative stress when compared to sedentary patients [99].

### Navigating the Controversy: Antioxidant Supplementation in Male Infertility

In the recent years, and in the process of exploring idiopathic male infertility, oxidative stress has emerged as a pivotal culprit, which is defined as imbalance between the aerobic life byproducts, namely ROS and the antioxidant capacity. Such imbalance is commonly encountered in infertile males suffering from various inflammatory conditions, infections, and hazardous exposures [100]. In attempt to reverse the altered redox biology, aiming to minimize the hazardous oxidative stress, the role for antioxidant therapy emerges. A Cochrane review studying 6,264 infertile patients who were included in 61 studies, concluded that live birth in patients receiving antioxidant therapy may be 14%-26% compared to 12% with placebo (OR 1.79, 95% CI 1.20 to 2.67, *p* = 0.005, low-quality evidence), yet in the context of clinical pregnancy, the rate may increase to reach 12%-26% in patients receiving antioxidant therapy compared to 7% in patients with placebo (OR 2.97, 95% CI 1.91 to 4.63, *p* < 0.0001, low-quality evidence), but here was no significant different between placebo and treatment when it comes to miscarriage rates [101]. A study by Steiner et al., published one year later demonstrated contradictory results, stating that when comparing impact of antioxidant therapy on infertile males, no improvement in sperm parameters and DNA integrity was observed when compared to patients receiving placebo [102]. The discrepancy between these results could be attributed to using data from low quality RCTs, or those with high risk of bias. On the other hand, a systematic review and meta-analysis by Agarwal et al., studying the impact of antioxidant therapy from data derived from 45 RCTs involving 4,332 infertile patients, a significant increase in sperm concentration (*p* < 0.01), progressive motility (<0.01) and normal morphology (*p* < 0.01) in association with increased pregnancy rate (*p* < 0.01) was observed, yet no significant impact on live birth (*p* = 0.64) or miscarriage rate (*p* = 0.98) was noted [103].

### Gonadotropins in Male Infertility: Unraveling the Evidence

Spermatogenesis is a complex process that is regulated by the central control governed by the hypothalamic-pituitary axis, through the release of LH and FSH, which govern the androgen and sperm production respectively. In the context of male infertility associated with hypogonadotropic hypogonadism, treatment using gonadotropin replacement therapy is considered crucial as demonstrated by Ortac et al., where restoration of spermatogenesis and pregnancy reached approximately 86% and 67%, respectively [104]. In a Cochrane review including six RCT involving 456 patients, gonadotropin therapy was associated with increased pregnancy rate when compared to counterpart with placebo (16% vs 6%), emphasizing on effective role of gonadotropins in the management of male infertility [105]. FSH is a crucial gonadotropin hormone that serves to induce and maintain spermatogenesis through its crucial supportive role on Sertoli cells, and subsequent deficiency can be associated with negative impact on male fertility, necessitating restorative interventions. In the context of abnormal semen parameters in patients with idiopathic male infertility, and normal serum FSH, it has been proposed that adjunct FSH therapy could be potentially considered, with response being predicted according to level of FSH receptor gene polymorphism [106]. In the most recent European Association of Urology guidelines, a weak recommendation emerged, which cautiously advised with the role of FSH therapy in ameliorating spermatogenesis in selected group of male patients presenting with idiopathic oligozoospermia and normal FSH levels. Furthermore, being inclined to use higher FSH doses, exogenous FSH therapy is anticipated to demonstrate an improvement in DNA fragmentation index, AMH and Inhibin levels [1]. These recommendations are supported by some evidence suggesting a role of FSH therapy in improving sperm parameters and sperm DNA fragmentation (SDF) index in patients with idiopathic male infertility and normal FSH levels. A study by Colacurci et al., demonstrated that using 150 IU recombinant FSH every other day for three months, in males of infertile couples with elevated DNA fragmentation index, resulted in improved SDF in 67% of subjects [107]. Regarding patients with idiopathic non-obstructive azoospermia, there is little evidence FSH therapy appears to improve sperm retrieval rates, however, the general evidence supporting this rational of treatment in the management of non-obstructive azoospermia is limited [108].

### Comparative Analysis of Fresh and Frozen Sperm in ICSI: Unraveling Birth Outcomes

In the realm of ART, the debate using fresh vs. frozen sperm seems unravelling. In a comparative study by Wu et al., examining samples used by 317 patients with normal spermatogenesis and undergoing ICSI, no difference was revealed in implantation, pregnancy, miscarriage and live birth rates (*p* < 0.05), and in spite of the higher incidence of neonatal low birth weight using the frozen vs. fresh sperm samples (20.91% vs. 8.49%, *p* < 0.05), multiple logistic regression demonstrated that pregnancy status (single vs twin, *p* < 0.01) is the variable most associated with lower birth weight rather than the sperm status (fresh vs frozen, *p* > 0.05) [109]. Conversely, a study by Cai et al., studying 436 ICSI related pregnancies using sperm from patients with OA, used in a fresh vs. frozen state, revealed that the use of frozen sperm was associated with higher incidence of low birth weight [110]. Furthermore, systematic review and meta-analysis by Liu et al., examined ICSI outcome of 20 retrospective studies, which demonstrated higher pregnancy rate using fresh vs frozen epididymal sperm (44.1% vs 36.6% *p* < 0.05) [111]. A systematic review on 26 articles by Amer et al., studying the outcome in NOA patients after using fresh vs frozen sperm samples, demonstrated similar fertilization and clinical pregnancy rates [112]. Finally, in a retrospective study on couples using donor eggs, fertilization rates did not differ between fresh vs. frozen sperm (74.8% vs. 68.6%, *p* = 0.13), yet other parameters demonstrated higher outcomes in fresh vs frozen samples, including pregnancy rate (76% vs 67%) and implantation rate (64% vs 36%, *p* < 0.04). Conversely, significantly higher miscarriage rate was demonstrated in frozen vs fresh samples (33% vs 5.9% *p* = 0.013) [113].

## Controversies and Limitations

### Quality of evidence

The diagnostic work up of idiopathic male infertility relies on solid evidence concerning the importance of an accurate investigation of medical history, physical examination, basic semen analysis and hormonal evaluation. However, controversies still exist in specific topics such as the role of sperm DNA fragmentation, the best candidate for ROS measurement and empirical treatment of idiopathic infertile men. Most of the published studies, for instance, have investigated the association between SDF, ROS and semen quality. Very few, instead, have looked at pregnancy outcome which is the most important outcome for physicians specialized in reproductive medicine. Therefore, future studies should address this gap.

## Future directions

Due to the limitations of standard semen analyses, artificial intelligence (AI) has been integrated into male infertility management. For example, a machine learning (ML) model effectively predicted subsequent improvements in sperm parameters in 87% of men (area under the curve [AUC] = 0.72) following varicocele repair [114]. Another recent advancement involves AI methods that offer objectivity and suitability for analyzing video images, with the potential to enhance intracytoplasmic sperm injection (ICSI) by assisting clinicians in objectively selecting the optimal sperm. Morphological assessment models based on unstained sperm images are currently under development to enhance the ICSI technique by enabling real-time classification of images [115]. Additionally, AI has been utilized to construct predictive models for estimating sperm quality or sperm extraction in azoospermic men with non-obstructive azoospermia (NOA) [116,117]. Despite these advancements, the limited availability of data regarding the role of AI in male infertility means that these models are not yet widely employed in clinical practice.

Next-generation sequencing has contributed to the identification of several unknown genetic alterations in the context of male infertility and azoospermia including FANCA, PLK4, WKN3, MEI1, ADAD2, and TEX11 [115]. Likewise, epigenetic markers have been proposed as new predictors of positive sperm retrieval in NOA men [115]. For instance, ESX1 transcript was identified in approximately 95% (62 out of 65 samples) of males with the presence of spermatogenesis in testicular tissue [118]. Seminal plasma proteomics is another emerging field in male infertility. Differences in seminal plasma proteomic profiles have been identified between NOA and infertile men as well as in patients with varicocele [115]. Finally, several studies have explored radiomics as an innovative method to predict sperm parameters, however, with the implementation of computational images this tool will certainly help clinicians in understanding and treating idiopathic male infertility.

## Conclusion

Idiopathic male infertility is still the most complicated topic for clinicians from a diagnostic and therapeutic perspective. The prevalence of this conditions is strongly related to the quality of the baseline investigation of each infertile men and the availability of diagnostic tests. To comprehensively evaluated idiopathic infertile men, a detailed medical history and an extensive physical examination are mandatory to investigate potential causes of infertility. Similarly, besides standard semen analysis, advanced examinations, such as SDF and ROS measurement, are becoming of clinical relevance to assess fecundability potential and to guide MFI treatment. In terms of diagnostic tools, scrotal ultrasound is important to role out testicular masses and to classify varicocele severity; genetic investigations should be performed in men with severe oligozoospermia or azoospermia. Epigenetic changes have demonstrated to have a role in sperm production and a prognostic value in fertility outcomes, but are only investigated in research setting, since their application in clinical practice is still debated. Treatment of IMI is a controversial topic. Antioxidant treatment has been found to be a valid option to counteract ROS action, therefore it is widely used despite inconsistencies in terms of compounds and duration of treatment. Furthermore, gonadotropins are suggested in IMI to improve sperm quality and SDF, but high-quality studies are needed to identify the best candidate for this option. Given the limited ability of current diagnostic tool to capture the pathophysiological mechanism underlying IMI, future tests, likely based on artificial intelligence, are needed to uncover this important field of male reproduction.
